# Treatment of Parturient Paresis by Iodide of Potash1Paper read before the annnal meeting of the Pennsylvania State Veterinary Medical Association, March 6, 1901.

**Published:** 1901-06

**Authors:** E. Mayhew Michener

**Affiliations:** Colmar, Pa.


### THE JOURNAL

OF

### COMPARATIVE MEDICINE AND VETERINARY ARCHIVES.

VOL. XXII. *JUNE, 1901.* No. 6.

### TREATMENT OF PARTURIENT PARESIS BY IODIDE OF POTASH.[1]

BY E. MAYHEW MICHENER, V.M.D.,

COLMAR, PA.

THE purpose of this paper is to furnish a summary of the results which have followed the use of iodide of potash in the treatment of parturient paresis.

As the drug is administered by its infusion in sterile solutions into the udder of the cow, some special apparatus is required. The apparatus I use may be made as follows: Take five feet of best rubber tubing of calibre to fit tightly over an ordinary milking-tube, which is fastened to one end of the tube, while to the other end is fastened a small glass funnel about three inches in diameter at its wide end. In use the milking tube is inserted into the teats in the usual manner, and the iodide of potash solution is poured into the funnel, which is kept elevated so that the solution flows into the udder by gravity.

The amount of iodide of potash required for each injection has been named by some at two and one-half drachms, but there is no reason why the size of the dose should not be governed by the size or weight of the animal and the severity of the attack. I have repeatedly given as much as four drachms with the most desirable results following, and am of the opinion that severe cases starting soon after calving should receive the maximum dose. To two-and-one-half drachm doses one quart of sterile water at about 100° F. should be used as a solvent for the iodide; larger doses have been given with corresponding increase in the amount of

[1] Paper read before the annual meeting of the Pennsylvania State Veterinary Medical Association, March 6, 1901.

water. It is clearly of the greatest importance that the water used for this purpose be absolutely sterile and free from sediment; freshly distilled water is the ideal form, but as it is at times difficult to obtain, and as old distilled water is unsafe, I have during the past year used freshly boiled water from the farm kitchen, filtered, if necessary, through ordinary sterilized cotton in a glass funnel. The iodide of potash should be carefully weighed and placed in sterile bottles and kept sealed until the time for use arrives. The bottles should be carefully labelled with the amount contained, as this saves time and rehandling when time is valuable. A three-pint porcelain pitcher, or, better still, a large glass graduated measure, is the most convenient vessel in which to dissolve the iodide. The advantage of a graduated measure is that exactly the amount injected can be told at a glance, and the amount of solution can be in this way evenly distributed to the four quarters of the udder.

The preparation of the udder to receive the injection requires the following: Remove all milk; wash the udder thoroughly with abundance of warm water and soap; especial care should be given to have the orifices of the teats perfectly clean; keep the udder from contact with the floor or bedding by the use of perfectly clean cloths. Immediately before the injection is begun the teats should be wetted with a solution of bichloride of mercury 1 to 2000. The apparatus, before described, having first been immersed in boiling water for at least one minute for the purpose of sterilization, is now removed and the milking tube inserted into one of the teats and one-fourth of the solution allowed to flow into that quarter; then the tube is changed, and in this manner each quarter receives its due amount. Two persons are required to make the injection, but a third, to assist in holding the patient's leg out of the way, is of great advantage. The injection once finished the time is noted, and in from twenty to thirty minutes the teats should receive a thorough milking, thereby removing any of the solution which may not be absorbed. The milking should be repeated at least every hour following, and the second injection, applied with the same precautions of preparation as before named, should be made in from eight to twelve hours according to the needs of the cases. From recent experience the repetition of the dose every eight hours is indicated.

In nine-tenths of the recoveries only two injections were given, and in no case were more than four injections given. The injection is discontinued as soon as the patient regains the use of the limbs sufficiently well to stand. In a small number of the cases, in which paralysis of the posterior limbs remained after the acute symptoms of the disease had passed, the continued use of the treatment has not been followed by results that would seem to indicate its further value in such cases. In the treatment of the last nineteen cases no medicine whatever was used aside from the amount of iodide named. In cases living but unable to rise at the end of the fifth day of sickness, other drugs have been tried with only ordinary success. During my early employment of the treatment the regulation dose of a saline purgative was given each case, but I have now discarded its use entirely, and the results are considerably better. There is reason for believing that the use of purgatives as generally administered is unnecessary and not uncommonly harmful. The hard feces, if any, should be removed from the rectum by the hand or by copious injections of warm water with an occasional four ounces of glycerin added. The bowels invariably become active with the abatement of the acute symptoms. The administration of large doses of purgatives invites pneumonia; the same is likely to result from allowing water in too large quantities when the patient becomes able to drink; the muscles of the pharyngeal and laryngeal region are rendered more or less incompetent from paralysis and foreign bodies. Liquids and even solids do gain entrance to the bronchi in not a few instances, as autopsies have shown. When the patient desires water it should be given in small quantity and frequently; possibly four quarts at a time are enough, which can be repeated every hour until thirst is allayed.

The following results have followed the iodide treatment: Total number of cases treated, seventy-two; total number of recoveries, fifty-eight; total number died or destroyed as hopeless, fourteen.

Among those making recovery, two cows recovered from two attacks; the second attack following the first at the next calving; the second attacks were apparently of greater severity than the first, but the treatment was applied earlier on account of great watchfulness on the part of the owner.

The flow of milk following recovery is frequently below the amount of previous periods of lactation, but not uncommonly regains the usual amount after one or two months. In no case has complete loss of portions of the gland resulted in the animals which have recovered, but two cases died with symptoms of septic infection of the udder and lungs. This complication is not unknown under other forms of treatment, and can scarcely be charged to infection through the agency of the injection. I have also employed the treatment with the idea of prevention of the disease in animals thought to be predisposed, but as the number of cases so far is limited to six the value of its employment for such use is not known. The six cows escaped an attack and milked as well as usual.

As nearly as can be estimated treatment of the seventy-two cases above mentioned began at an average of four hours from the first symptom of the attack. In all but four cases the animal was found down, unable to rise, at the time of my first visit. Four cases were injected while still standing, but about ready to go down, which they invariably did; and it is worthy of note that in all four the recovery was rapid and complete, indicating, so far as it goes, the importance of the early application of the treatment. It might be mentioned that good nursing was very fairly carried out in almost all the cases mentioned. The cow was placed in a dry and level stall whenever possible, and much care was given by the attendants in keeping her in good position, so essential to the good treatment of any animal in the recumbent position, and of especial importance in this particular disease.

In my opinion the following points are of great importance in the treatment of parturient paresis:

1. Early detection and application of treatment.
2. Absolute cleanliness of udder, apparatus, and hands of the operator, and a sterile condition of the injection.
3. Care and attention to the position of the patient, and the administration of the injection at the proper time and of the right amount.

### Discussion.

Dr. George B. Jobson: Mr. President and Gentlemen: The subject is quite an important one, and has forced itself sometimes very unpleasantly on my attention. The general line of treatment which Dr. Michener adopts is pretty much the same as that which we all use—the iodide of potassium treatment. Previous to trying this treatment my practice was to administer a purgative, and give a hypodermic injection of digitalis and strychnine to stimulate the heart and restore intestinal peristalsis. When I commenced with the iodide of potassium I quit using hypodermic injections of digitalis and strychnine. Several of these cases fainted away and did not recover. Since then, while using the iodide of potassium treatment, I give one-half grain of strychnine and fifteen minims of digitalis hypodermically. I have found good results giving this every four hours in conjunction with iodide of potassium. The latter I use every two hours, using two and one-half drachms in sterilized water.

In this country, at least, the Jerseys are much more subject to the disease than any other kind of cattle. It may be that I have had more to do with

Jerseys, but I have found it so. In Scotland the Ayrshire breed of cattle is subject to the disease more than any other. There we have a breed of cattle that are called Highland cattle. I have never known one of these taking milk-fever. They allow the calves to suckle the cows and then wean them. I do not know whether any of you gentlemen have had any heifers take milk-fever. I have not had it with the first calves, but I have with the second calves. It generally occurs in cows with the third, fourth, or fifth calf. Blood plethora was the old theory of milk-fever, the supposition being that the maternal blood supplying the fœtus *in utero* being cut off from the fœtus after parturition caused congestion before relief could be obtained through the lactic system. Acting on this theory and to prevent this I tried bleeding. I have found it successful. I tell you, gentlemen, the best preventive is milking before calving. I have found that in almost every case an absolute preventive for milk-fever. The theory on which it is based is reasonable. The disease is caused by auto-infection, an absorption of the decomposed milk from the previous term of milking. Before it has a chance to absorb in the system you take it away by first milking before the cow calves. I would advise anyone who has any reason to think that the cattle under their charge are predisposed to milk-fever to milk out, and I think you will be abundantly satisfied with the results. Cure is a very good thing. "An ounce of prevention is worth a pound of cure."

Dr. Porter: Use a wide-mouth bottle. Make your preparation sterilized, of course, and cork it up, and when you use it set it in a pail of water; get the right temperature, attach your tube, and turn it upside down. There is then no danger of infection from outside influences.

Dr. Jobson: When the cow has a full bag that is the time to milk the cow. That is the time to commence milking her.

Dr. W. H. Ridge: Mr. President: I do not know that I have anything to add. I am in favor of the iodide of potassium treatment. I think the results are better than any line of treatment I have ever tried, but I cannot get as good results as Dr. Michener. I have kept a record of twenty-three cases, and fifteen of those recovered. Some of the others had to be killed or died. The gentlemen spoke about milking cows out as a preventive. I have had several cases of milking out before calving which were followed by parturient paresis, so that I do not think that that is a preventive for the complaint.

The point that I would like to be enlightened on more than the treatment of parturient paresis in the first stage is the paralysis of one hind leg after a few days, after they commence to eat and milk and ruminate, and seem to be in a fair way to recovery, but yet unable to rise. These cases I find much more difficult to recover than the majority of cases. You see bad cases which you think are going to die, and in a few hours they are on their way to recovery; and other cases linger on for several days and probably weeks and have to be killed. I know of several cases where the animals had sloughing of the hoof; the skin sloughed off, and it looked like ergotism. This is attributed to allowing the cow to be too long on one side, cutting off the blood-supply by pressure. It has occurred where they have been turned every three hours. I have been unable up to the present to know how to prevent this occurrence.

Dr. Jobson: How long before calving do you milk them?

Dr. Ridge: About three days. I do not advise a cow being milked longer before being calved. I would say about three days before calving, if I could judge the time, and then I want it milked night and morning.

Dr. Jobson: You milk her up before calving?

Dr. Ridge: Yes.

Dr. Jobson: I have had several cases before parturition, and that is what I want to know—how long you milk beforehand to prevent it. I can count four cases.

Dr. Sallade: I want to ask Dr. Jobson a question. I suppose nearly all have had a similar experience with parturient apoplexy. I want to ask him what are the predisposing signs of parturient apoplexy?

Dr. Jobson: I may be wrong about this theory of mine, that certain families are predisposed to milk-fever, but I have followed this thing up very closely with regard to the breeding of heavy milkers, and have found that generally the daughter has followed in the footsteps of the mother. A man may have a theory which impresses itself on his mind, and it has done so in my case. I can name certain cases where they died from milk-fever and where their daughters died from milk-fever. If there is anything in it I do not know. These reproduce the same characters as the dams had. The offspring—and it may be the same as in tubercular consumption—have a predisposition toward the disease. It may be an inherited predisposition.

Dr. Leonard Pearson: I think another important thought is that milk-fever is unquestionably due to activity of the udder. It is not due to plethora, because beef cows do not have milk-fever. No matter how fat they are when they calve, they do not contract this disease. One way of preventing milk-fever, which is found to be effective in practice, is to starve the cows or feed them on small quantities of food before they calve. This lessens the activity of the udder. The less you feed a cow the less she will milk, and the less she is predisposed to milk-fever; and yet you will see cows contract milk-fever when they are eating grass in the summer time. If the cow is on rich pasture she will get it the same as if she was fed on rich food in the stable. If she is on scanty pasture or on a small quantity of food she does not get milk-fever, because the udder is not stimulated. The proof of the soundness of this theory of milk-fever is shown by the results of the Schmidt-Kolding treatment.

The circular I sent out some two years ago to the veterinarians of Pennsylvania in reference to this subject showed that 75 per cent. of the cows treated in this manner recovered. The Danish statistics claim 80 per cent. of recoveries; a recent German writer claims 82 per cent. of recoveries, and I have no doubt that we will reach this figure in time; but even at 75 per cent. the results are much better than ever before, and the soundness of the theory upon which this treatment is based is indicated.

The President: Gentlemen, we cannot stop here. This matter interests everyone, especially in the agricultural districts and dairy districts.

Dr. Porter: About three or four years ago I began using it as a preventive. I have not the statistics, and I do not know of a case of parturient apoplexy following treatment, for two or three days before calving, with nux vomica. There was one dairy especially where there was a very high feeder, used to have lots of trouble, but has not had a case of apoplexy since using the nux vomica treatment, drachm doses three times a day.

A MEMBER: I would like to ask Dr. Pearson if diminishing milk-producing foods before calving would not have a tendency to reduce the quantity of milk after calving?

DR. PEARSON: I do not think so. A cow would be slower in coming to milk, but she would have just as much.

A MEMBER: Where cows are predisposed I think these weaknesses are transmitted. Would it not be a good plan where we fear milk-fever after calving to milk the udder out clean several times and at least once to give her the iodide treatment beforehand?

DR. PEARSON: Yes, I think that would be a very valuable precaution, and if there are special reasons to fear milk-fever I do not see why that should not be done. It is a very valuable thing.

DR. CLARENCE MARSHALL: I had a little experience last year with milk-fever that made me a believer in what Dr. Jobson spoke of, viz., milking cows out before calving. One herd that I visited frequently had five cows with milk-fever in succession, and four of them died. I blamed the superintendent for overfeeding. I left instructions not to feed the same rations when dry as when in milk. The superintendent advised me that they did not feed as much, but fed from the floor, and they could get food on either side of them, as there was no partition between the feeding-places. I reasoned that if it were due to auto-intoxication the proper thing to do was to get rid of the colostrum. I asked him to milk the cows out as soon as the udder began to get distended. This was done, and they have had no milk-fever since, and that has been a year. They feed the cows as before. I thought this was an original idea, and I bragged about it to an old practitioner, and he said it was an old idea. I think there is enough in it to advise people to milk cows out before calving, if the udder is very much distended and you fear milk-fever The last four cases treated with iodide of potassium were bad cases. Three of them got well.

DR. RIDGE: Sometime ago there was at the Keystone Association a Mr. Detrick who gave a report of cases of milk-fever in his herd and presented the report that Dr. Rayner gave him a method that would stop it entirely in his herd. I think Dr. Rayner could give us some enlightenment. The point is this: to stop all hay and large quantities of food before calving. I have used that way to advantage. I know one herd that was afflicted very much with parturient paresis, and since they adopted that and put the cows on the floor, where they could not eat hay, and on a starvation diet it stopped the disease. I also notice that cows in herds that were given a good saline purgative as soon as they were fresh they had very little trouble in the matter.

DR. RAYNER: My idea of this milk-fever disease is to stop your extreme feeding and give plenty of exercise. You will have no milk-fever. It is where you have a good cow and want it to come in in excellent condition. They feed to extremes, with no exercise, and the extreme labor of delivery in calving produces your trouble. That is my experience. Cows half-fed, with plenty of exercise, very seldom have milk-fever. Prevention is better than cure any time, and that is my method.

DR. LOWE: Mr. President: I do not know that I have anything to add to what has already been said. I have been very much interested in the discussion. I believe, of course, that the prevention is more valuable than the cure. The restricting of the diet and the exercise, as Dr. Rayner records, is the key to the situation as regards prevention, and I think the point Dr. Pearson brought out that the disease is due to the activity of the udder rather than to plethora, as we used to think, is probably the correct idea in regard to it. I have nothing more to add on the subject.

DR. RECTENWALD: We find tyrotoxicon in fish, in milk, in bread. I think we should get rid of that milk as much as we can. I have seen it in cows in the field and in stables, and I do not know of any other preventive as good as to get rid of that milk in the udder that brings out tyrotoxicon poison.

DR. H. ALLIS: As a preventive I have found people take cows off from grass and put them in a stable for a few days before calving. Those cows are all plethoric animals and deep milkers, and invariably I have had good results from removing them from the pasture, putting them into the stable, and feeding lightly. The idea ought not to be to reduce the flesh, but reduce the activity of the udder. I think that is the preventive. Light exercise and milking out, too, I have found to be very essential.

A MEMBER: There is one question I would like to know. What action this milking-out business has on the meconium of the calf. I find when a cow has been milked out thoroughly the meconium of the new-born is not moved readily. I would like to hear some experience brought out on it.

DR. JOBSON: That is a little out of order. We are treating the cow; we are not treating the calf. But, of course, it might be a good thing, but I do not see why we cannot have that overcome by giving the calf small doses of castor oil, or take a tablespoonful of brown sugar in a half-pint of water; but the fact is this: of the two ills choose the lesser. I would rather lose the calf than lose the cow.

Further discussion was entered into by Drs. J. C. Michener, Ross, Beeber, Tower, Collins, McNeal, Benner, Irons, and others, deviating somewhat from the subject germane.

